# Odontogenic causes complicating the chronic rhinosinusitis diagnosis

**DOI:** 10.1007/s00784-020-03384-4

**Published:** 2020-06-04

**Authors:** Annina Wuokko-Landén, Karin Blomgren, Anni Suomalainen, Hannamari Välimaa

**Affiliations:** 1 Faculty of Medicine, University of Helsinki, Helsinki, Finland; 2 Department of Otorhinolaryngology, University of Helsinki and Helsinki University Hospital, Helsinki, Finland; 3 HUS Medical Imaging Center, Department of Radiology, University of Helsinki and Helsinki University Hospital, Helsinki, Finland; 4 Department of Virology, University of Helsinki, Helsinki, Finland; 5 Department of Oral and Maxillofacial Surgery, University of Helsinki and Helsinki University Hospital, Helsinki, Finland

**Keywords:** Diagnosis, Paranasal sinus diseases, Maxillary sinus, Dental pulp diseases, Pathology, Retrospective study

## Abstract

**Objectives:**

Chronic rhinosinusitis (CRS) frequently stems from a dental origin, although odontogenic sinusitis (OS) remains underdiagnosed amongst different professionals. This study aimed to explore how often odontogenic causes are considered when diagnosing CRS.

**Materials and methods:**

Patient records from 374 new CRS patients treated at a tertiary-level ear, nose, and throat (ENT) clinic were selected. Entries and radiological reports were assessed exploring how often dentition was mentioned and OS was suspected, how often radiologists reported maxillary teeth, and how commonly typical OS microbial findings and unilateral symptoms occurred.

**Results:**

Although 10.1% of the CRS diagnoses were connected to possible dental issues, teeth were not mentioned for 73.8% of patients. Radiological reports were available from 267 computed or cone beam computed tomographies, of which 25.1% did not mention the maxillary teeth. The reported maxillary teeth pathology was not considered in 31/64 (48.4%) cases. Unilateral symptoms associated with apical periodontitis (OR = 2.49, 95% CI 1.27–4.89, *p* = 0.008). Microbial samples were available from 88 patients, for whom *Staphylococcus aureus* was the most common finding (17% of samples).

**Conclusions:**

Odontogenic causes are often overlooked when diagnosing CRS. To provide adequate treatment, routine assessment of patient’s dental history and status, careful radiograph evaluation, and utilization of microbial findings should be performed. Close cooperation with dentists is mandatory.

**Clinical relevance:**

Dental professionals should be aware of difficulties medical professionals encounter when diagnosing possible OS. Thus, sufficient knowledge of OS pathology is essential to both medical and dental professionals.

## Introduction

Chronic rhinosinusitis (CRS) is a common disease affecting approximately 5 to 15% of the general population both in Europe and the USA [1]. CRS has a considerable impact on patient quality of life [2] and healthcare costs worldwide [3]. Patients with chronic sinonasal symptoms report pain, sleep problems, and even depression more often than healthy controls [4]. Because the maxillary teeth lie within close proximity to the maxillary sinuses and because dental infections affect a large number of patients, odontogenic sinusitis (OS) represents a common form of both acute rhinosinusitis (ARS) and CRS. However, OS is often overlooked [5], and in contrast to OS, no generally accepted diagnostic criteria exist.

In over 60% of cases, odontogenic chronic maxillary rhinosinusitis is caused by iatrogenic factors, such as tooth extractions and root canal treatments. The first and second maxillary molars are the most common origins of the disease [6, 7]. Managing OS requires treating both the dental issue and sinus condition, and sinus surgery is often required [8]. Sinonasal surgery effectively manages CRS patients for whom medical treatment has failed [1, 9, 10]. Unrecognized odontogenic infection can, however, lead to surgical failure [11].

The dental pathology underlying rhinosinusitis is often missed in computed tomography (CT) scan reports [12], potentially delaying correct diagnosis and treatment. The exact pathophysiology of OS remains under investigation [13]. Yet, bacterial biofilms may play a crucial role, and there seems to be a difference in the microbiology of CRS and CRS of odontogenic origin [14].

No single symptom is specific to OS, but patients often have unilateral symptoms and a foul-smelling nasal discharge. OS is usually recalcitrant to medical therapy [15]. Moreover, in addition to the maxillary sinuses, OS can affect other paranasal sinuses [16]. An accurate diagnosis requires simultaneous attention to several factors, namely, the patient’s dental status and history, radiological findings, and microbial findings and symptoms.

In this study, we sought to explore how often OS is considered when diagnosing CRS patients and to determine the proportion of possible chronic OS patients in a tertiary healthcare setting. We also evaluated the routine diagnostic tools available and findings that could lead to OS diagnosis, such as unilateral symptoms, radiographs and their reports, and microbial findings.

## Materials and methods

### Patients and variables

We retrospectively selected all 1690 CRS patients who visited the Department of Otorhinolaryngology at Helsinki University Hospital (HUH) in 2013. Patients were identified through a search for International Statistical Classification of Diseases and Related Health Problems 10th Revision (ICD-10) diagnosis codes J32 entered into their patient records. Diagnosis was determined by the ear, nose, and throat (ENT) specialist or resident treating the patient. ICD-10 codes included here were J32.0 (chronic maxillary sinusitis), J32.1 (chronic frontal sinusitis, except clearly isolated ones), J32.2 (chronic ethmoidal sinusitis), J32.4 (chronic pansinusitis), J32.8 (other chronic sinusitis), and J32.9 (unspecified chronic sinusitis). Each patient had one or more of these diagnosis codes.

Only patients whose disease was diagnosed as chronic in our clinic for the first time in 2013 were included. We excluded patients with previous sinus surgery.

The data were collected from electronic records: patient record entries, radiological reports, and laboratory databases. Some patient background details were verified from paper documents. Patient background characteristics included sex, age, other diseases, allergies, and smoking status. The number of suspected OS diagnoses according to the entries was recorded along with any considerations of teeth and oral mucosa when diagnosing CRS.

The number of radiographs (taken at HUH or at the referring unit) where maxillary teeth could be adequately examined and which were used in CRS diagnostics were recorded. These radiographs consisted of panoramic tomographies (PTG), sinus CTs, and sinus cone beam computed tomographies (CBCT). The electronic database lacked some radiological reports (primarily radiographs taken at the referring unit); these were not accessible for our study. The radiologists’ comments on the maxillary teeth were also recorded. The association between reported radiological dental pathology, reported radiological apical periodontitis, and the patient’s unilateral symptoms were analysed.

If the patient had samples taken for microbiological or histological analysis, the findings were recorded from the hospital laboratory database. The duration of symptoms and their possible unilaterality were observed.

### Statistical analysis

The associations between variables were analysed using chi-squared test or the Fisher’s exact test. Binary logistic regression was used to evaluate the association between unilateral symptoms with pathological dental findings and apical periodontitis according to radiological reports. We report our results as odds ratios (ORs) with 95% confidence intervals (CIs). Statistical analyses were performed using IBM SPSS Statistics, version 24.0 (IBM Corp., Armonk, NY), and we considered *p* < 0.05 as statistically significant.

## Results

### CRS patients

A total of 374 CRS patients met the inclusion criteria. CRS was linked to a possible odontogenic cause in 38 patient record entries (10.1%). Table 1 summarizes the background characteristics of the patients. During 2013, 145 (38.8%) patients underwent and 82 (21.9%) were scheduled for sinus surgery. Almost one-fourth (90 patients, 24.1%) reported unilateral sinus symptoms. Symptoms persisted for < 1 year in 111 patients (29.7%), > 1 year in 36 (9.6%), and several years in 179 (47.1%). Patient records lacked this information in 48 cases.

**Table 1:** Characteristics of 374 patients with a diagnosis of chronic rhinosinusitis

Characteristic	*n* (%)
Gender	
Female	257 (68.7)
Male	117 (31.3)
Mean age, in years (SD)	44.5 (17.3)
Smoking status	84 (22.5)
Allergies	152 (40.6)
Asthma	67 (17.9)
Disease predisposing to infections	49 (13.1)
Disease of lower respiratory tract other than asthma	6 (1.6)
Pregnancy	3 (0.8)

*SD* standard deviation

Teeth were not mentioned in 276 (73.8%) patient records. In 10 cases (2.7%), the disease was described as ambiguously odontogenic (Table 2). The characteristics of these 10 most likely OS patients appear in Table 3. Seven of ten patients complained of unilateral symptoms. All radiological reports available (7 of 10) indicated pathological dental findings in the maxillary teeth, and in 5 of 6 microbial samples with growth bacterial findings typical for oral flora emerged.

**Table 2:** Possible OS diagnoses and observations of teeth and mouth in 374 chronic rhinosinusitis patient records

Observations from patient records	*n* (%)
Possible OS	38 (10.1)
Suspected OS	28 (7.5)
Suspected OS and patient referred to a dentist	18 (4.8)
Suspected OS without further action	10 (2.7)
OS described as odontogenic	10 (2.7)
Teeth mentioned	98 (26.2)
Teeth not mentioned	276 (73.8)
Teeth examined (for example, by tapping)	13 (3.5)
Oral mucosa examined	273 (73.0)

*OS* odontogenic sinusitis

**Table 3:** Characteristics of 10 patients with odontogenic sinusitis according to patient records

	Age	Sex	Symptoms and their duration	Microbial findings	Radiological examinations	Pathological maxillary teeth findings in radiological reports
1	72	Female	Unilateral, < 1 year	Aerobic mixed flora Anaerobic gram-negative rods^a^	Plain sinus radiograph CBCT	NA
2	49	Male	Unilateral, NA	Aerobic mixed flora *Streptococcus anginosus* group^a^ Anaerobic gram-negative rods^a^	CT	Yes
3	46	Female	Unilateral, > 1 year	Aerobic mixed flora Anaerobic gram-negative rods^a^	CT PTG	Yes
4	67	Male	Unilateral, several years	Anaerobic gram-negative rods^a^	PTG	Yes
5	30	Male	Bilateral, < 1 year	No sample	CT	Yes
6	47	Female	Unilateral, several years	No sample	CBCT	Na
7	71	Female	Unilateral, > 1 year	No growth	CT	Yes
8	61	Male	Unilateral, < 1 year	No sample	CT	Yes
9	81	Male	Bilateral, NA	Coagulase-negative staphylococci *Staphylococcus aureus* *Propionibacterium acnes*	CT	Yes
10	66	Male	Bilateral, < 1 year	*Staphylococcus aureus* *Streptococcus anginosus* group^a^	NA	NA

^a^Microbial findings typical for oral microbiota

*CBCT* cone beam computed tomography, *CT* computed tomography, *PTG* panoramic tomography, *NA* not available

### Radiological examinations

**Fig. 1:**
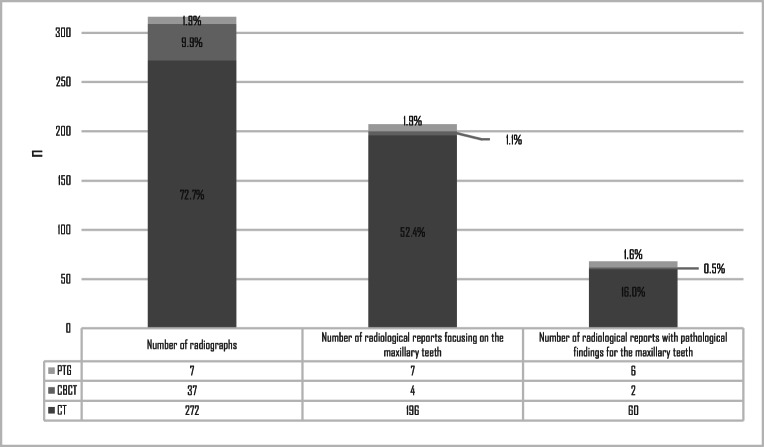
The number and distribution of radiographs for which the maxillary teeth could be examined (*n* = 316). Note that the maxillary teeth and pathological findings were based on the radiological reports available. Percentages represent the proportion of all 374 patients. PTG panoramic tomography, CBCT cone beam computed tomography, CT computed tomography

Most patients (353/374, 94.4%) underwent a radiograph at HUH or at the referring unit. Maxillary teeth could be examined in 316/374 (84.4%) radiographs (Fig. 1). These radiographs were taken from 312 patients (four patients had both PTG and CT radiographs). The radiographs where maxillary teeth could be examined are presented in Fig. 1. A total of 67/267 (25.1%) CT and CBCT radiological reports available did not mention the maxillary teeth at all. Nearly one-third (32.9%) of the reports that mentioned the maxillary teeth reported pathological dental findings. According to the radiological reports, 64 patients (17.1%) had pathological maxillary dental findings. In 31/64 cases (48.4%), this information was not considered or mentioned to the patient.

**Fig. 2:**
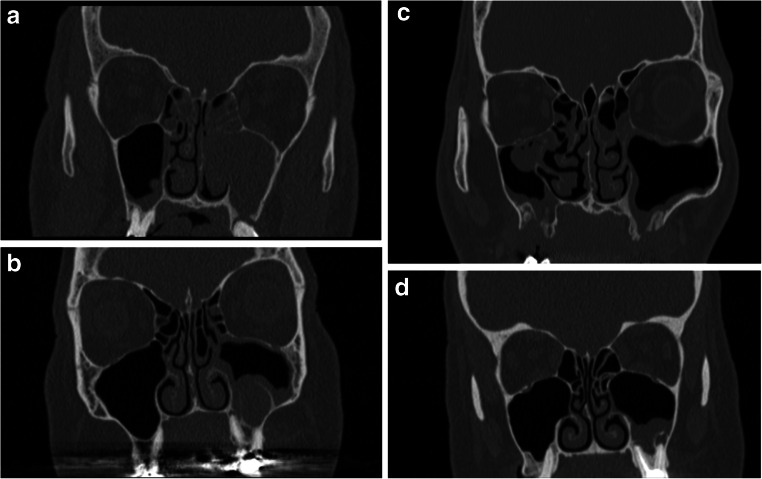
Coronal computed tomography views of four likely chronic odontogenic sinusitis patients. The pathological maxillary teeth findings were examined based on the radiological reports. **a** Apical periodontitis in the left first maxillary molar. An oro-antral fistula following extraction of the left second maxillary molar and an infected, unerupted left third maxillary molar connected to the previous fistula (not shown). **b** Deep filling and untreated roots in the left first maxillary molar. A cystic lesion extends from the buccal roots to the sinus and a small apical periodontitis surrounds the palatal root. **c** Bilateral oro-antral fistulas following extractions of the left first and third maxillary molars and right second and third maxillary molars. Defects in the maxillary sinus floor bone, maximum size 15 mm × 9 mm on the right side and 7 mm × 9 mm on the left side. **d** Apical periodontitis in all of the roots of a root canal treated left first maxillary molar and bone defects from the buccal roots to the maxillary sinus floor

Unilateral symptoms did not associate with the reported pathological dental findings (OR 1.75, 95% CI 0.96–3.17, *p* = 0.066). Unilateral symptoms were observed in 42 patients and associated with reported apical periodontitis (OR 2.49, 95% CI 1.27–4.89, *p* = 0.008). Figure 2 illustrates the pathological maxillary teeth findings of four CRS patients likely to have chronic OS.

### Microbial culture, native microscopy, and sinus biopsy findings

Antral irrigation was performed on 92 (24.6%) patients and lavage of the maxillary sinus on 96 (25.7%) patients. In total, 88 patients had a microbial sample taken, from which 80 (90.9%) showed microbial growth. Each sample had on average 1.5 microbial isolates. Table 4 summarizes the microbial findings.

**Table 4:** Microbial isolates in 88 maxillary sinus samples from 374 patients with chronic rhinosinusitis

Isolates	*n* (% of 134 isolates)	*n* (% of 88 samples)
Aerobes	106 (79.1)	77 (87.25)
Unspecified aerobes (mostly normal flora)	36 (26.9)	36 (40.9)
Gram-positive cocci	35 (26.1)	31 (35.2)
Staphylococci	22 (16.4)	20 (22.7)
*Staphylococcus aureus*	15 (11.1)	15 (17.0)
Coagulase-negative staphylococci	7 (5.2)	7 (8.0)
Alpha-haemolytic streptococci	12 (9.0)	11 (12.5)
*Viridans* group streptococci^a^	5 (3.7)	5 (5.7)
*Streptococcus anginosus* group	4 (3.0)	4 (4.5)
Unspecified^a^	1 (0.7)	1 (1.1)
*Streptococcus pneumoniae*	7 (5.2)	7 (8.0)
Beta-haemolytic streptococci	1 (0.7)	1 (1.1)
*Streptococcus betahemolyticus* G	1 (0.7)	1 (1.1)
Gram-negative rods	35 (26.1)	30 (34.1)
Coliform rods	15 (11.2)	13 (14.8)
*Pseudomonas* species	6 (4.5)	6 (6.8)
*Pseudomonas aeruginosa*	5 (3.7)	5 (5.7)
*Pseudomonas stutzeri*	1 (0.7)	1 (1.1)
Other gram-negative rods	14 (10.4)	14 (15.9)
*Haemophilus influenzae*	11 (8.2)	11 (12.5)
*Moraxella catarrhalis*	3 (2.2)	3 (3.4)
Anaerobes	17 (10.4)	14 (15.9)
Gram-negative rods	10 (7.5)	9 (10.2)
Unspecified^a^	8 (6.0)	8 (9.1)
*Fusobacterium* species^a^	1 (0.75)	1 (1.1)
*Prevotella* species^a^	1 (0.75)	1 (1.1)
Gram-positive cocci^a^	2 (1.5)	2 (2.3)
Unspecified^a^	2 (1.5)	2 (2.3)
Gram-positive rods	3 (2.2)	3 (3.4)
*Propionibacterium acnes*	3 (2.2)	3 (3.4)
Anaerobic mixed growth^a^	2 (1.5)	2 (2.3)
Fungi	11 (8.2)	9 (10.2)
Fungal hyphae	7 (5.2)	7 (8.0)
*Candida albicans*^a^	2 (1.5)	2 (2.3)
*Aspergillus candidus*	1 (0.7)	1 (1.1)
*Aspergillus fumigatus*	1 (0.7)	1 (1.1)

^a^Microbial findings typical for oral microbiota

Twelve patients had fungal findings either in the microbial samples (culture or native microscopy), in sinus biopsies or in both. Histological diagnoses were available for 52 patients; all of these patients also had microbial samples. Five of the biopsies were suggestive of fungal infection, and two of these patients also had fungal findings (one sample with *Aspergillus fumigatus* and fungal hyphae, another with fungal hyphae alone) in the microbial samples. Four patients had fungal findings (one with *Aspergillus candidus* and fungal hyphae, and three with fungal hyphae alone) in the microbial samples but not in the histological diagnoses. In addition, *Candida albicans* was identified by culture in two samples.

## Discussion

Although OS is repeatedly mentioned as a common form of both CRS and ARS, the disease remains underdiagnosed even upon radiological examination. Our study primarily aimed to explore how often odontogenic causes are considered when diagnosing CRS at the tertiary healthcare level, and we found 38 (10.1%) patients that had aroused ENT suspicion of OS. Although a total of 64 patients had pathological maxillary teeth findings according to radiological reports, in 31 cases these findings were not considered during treatment or mentioned to the patient. Because any possible dental treatments were not performed in our clinic, these OS diagnoses could not be retrospectively verified. Estimates of OS incidence vary, mostly from 10 to 25% of all rhinosinusitis cases [12]. In our previous study of ARS patients using a similar study design, 59/385 (15.3%) patients were suspected of having a possible odontogenic cause [17].

The condition of the oral mucosa was often noted (for 73% of patients), although the teeth were rarely mentioned (26.2% of patients) in reports. This likely reflects the difficulty and uncertainty of evaluating the approximate condition of dentition encountered by ENTs. Restorative treatment, such as large fillings and crowns, dental prostheses and implants as well as missing teeth, can implicate an underlying dental pathology and should be acknowledged. Because chronic OS typically results from previous dental treatment or oral surgery, charting of the patient’s dental history is essential also for ENTs. Apical periodontitis may become and remain chronic without causing tooth pain and can arise from dental diseases such as caries and may not necessarily heal following root canal treatment [18]. The prevalence of apical periodontitis varies widely between countries and populations. In a broad national health study of more than 5000 Finnish adult PTGs, 27% revealed one or more apical periodontitis, most commonly in molars [19]. We found an association between reported unilateral symptoms and the radiological reports of apical periodontitis (*p* = 0.007). This strengthens the importance of unilateral findings as a possible sign for OS [20–23]. The presence of sinus mucosal thickening more likely accompanies with an apical periodontitis lesion larger in size [24].

In addition to apical periodontitis, conditions such as marginal bone loss, a loss of bone between the tooth and maxillary sinus for various reasons including post-extraction oro-antral communication, and procedures used to augment the alveolar process prior to the placement of dental implants should also be considered [23, 25]. Clinicians should also keep in mind that periapical and marginal destruction can cause basal mucosal thickening to varying degrees. These findings can also be indicative of a natural reaction to a low-grade infection from an adjacent tooth rather than sinusitis. It is also important to realize that periapical destruction does not always cause sinusitis, especially if bilateral sinusitis is observed [23].

Multislice CT is the standard 3-dimensional (3D) imaging method for the specific diagnosis of the maxillary sinus. CBCT offers a low-dose alternative to 3D imaging, and it was used in few cases in our study material [26–28]. Although CT and CBCT provide valuable information about dentition, teeth were not mentioned in 25.1% of the available radiological reports. In some cases, this shortage may simply result from a healthy condition or difficulties in maxillary teeth diagnoses because of artefacts or an incomplete view. Unfortunately, radiological reports often miss dental pathology, which is probably also true in our data. To avoid confusion, we recommend that radiologists always comment on the teeth, since the recognition of dental disease by a radiologist may play a significant role in subsequent treatment decisions [29].

In the radiological report, dental findings could be categorized as follows: (1) radiological findings referred to OS, (2) potential radiological findings referred to OS, and (3) no radiological findings referred to OS. In the two first situations, consultation of dentist should be mandatory, a consultation which demands follow-up. Additionally, the radiological examinations and radiological reports should be available to the dentists. Moreover, an oral and maxillofacial radiology specialists with expertise in this should be consulted whenever possible. In our tertiary clinic, both ENT and oral and maxillofacial radiologists are available.

More than half of the patients underwent or were scheduled for surgery. In addition, almost half of the patients had experienced sinonasal symptoms for several years. The surgical management of patients with sinonasal complications related to dental disease or treatment poses a significant challenge [8]. First-line sinonasal surgery followed closely dental treatment can result in the quicker resolution of sinus symptoms than vice versa [30]. Therefore, early OS recognition is essential to providing timely, adequate treatment and to avoiding unnecessary costs.

In our study, microbial culture samples were available from 88 patients (23.5%).The most common finding was *Staphylococcus aureus* (present in 17.0% of samples), often and abundantly found in CRS patients [31]. Whilst bacteria play an established role in ARS and acute infectious exacerbations, their role as initiators of CRS remain unclear [1]. Instead of traditional culture-based studies, culture-independent techniques are now often used to investigate the complex sinus microbiome, which can vary even between sinuses in the same individual [32]. Apparently, microbiome diversity decreases and anaerobic flora is enriched in CRS patients compared with healthy and allergic rhinitis subjects [33]. Additionally, there seems to be depletion of protective, commensal species and an overabundance of disease-causing organisms [32]*.*

We previously showed that in ARS patients, cultures are easily available for most patients and that microbial findings typical for oral flora are often found in microbial cultures and associate with unilateral symptoms [17]. Here, microbial culture were taken for less than one-fourth of the patients and only a few samples represented findings typical for oral flora. This result emphasizes the challenges in diagnosing OS in more chronic cases and underlines the importance of radiological imaging. Like ARS and CRS, acute and chronic OS appear to represent different entities with dissimilar pathology and microbiology. The microbial burden seems to be larger in OS than in CRS [34].

Root canal treatment materials misplaced in the maxillary sinus may favour the growth of certain microbes, including *Pseudomonas aeruginosa (P. aeruginosa)* and *Aspergillus* species [7, 35]. Several materials including sodium hypochlorite, calcium hydroxide, and gutta-percha are routinely used in root canal treatment. These materials may be unintentionally introduced into the maxillary sinus and cause inflammatory reactions. Overfilled root canal material can mechanically irritate the sinus mucosa, and certain chemicals have been shown to favour the growth of *Aspergillus fumigatus* [35]. *P. aeruginosa* associates with misplaced dental materials [7], and any sinus inflammation likely persists until removal of the foreign body [36]. Five of the study patients presented with *P. aeruginosa* and nine with fungal findings, of which two were *Aspergillus* species, two *Candida* species, and five fungal hyphae findings alone. Three patients had a fungal infection according to histological diagnosis, although not by culture or native microscopy. Our contradictory fungal findings from biopsies and microbial samples reflect the overall complexity of fungal sinusitis diagnostics.

Some limitations of this study should be considered. Our findings and conclusions are based on entries and radiological reports, representing the main weakness of this study. Additionally, our material consists of patients referred from primary healthcare and most apparent OS cases were presumably already diagnosed at the primary care level. Patient inclusion also relied only on ICD-10 diagnosis codes, and, therefore, the study patients may lack some CRS criteria. On the one hand, microbial sampling was not standardized due to different operators and techniques; on the other hand, the reported microbial findings show the actual microbiological information available to the clinician during the diagnosis and treatment of CRS patients.

Because OS patients often initially visit clinicians other than dentists first, a sufficient basic knowledge of dental pathology and treatment is essential for physicians as well as ENT and radiology specialists. Inquiring about a patient’s dental history should be routine in CRS diagnostics. Similarly, disseminating knowledge to dentists related to sinus disease and treatment is equally important. Moreover, all findings from microbial samples and radiographs should be carefully considered. We recommend cooperation across various specialists, reaching consensus on OS diagnostic criteria and creating clear treatment protocols to avoid misdiagnosis and treatment delays for this common disease.

## Conclusion

Odontogenic causes are overlooked when diagnosing CRS despite good diagnostic tools. Radiographs remain essential in OS diagnostics, although radiologists often miss dental findings. Unilateral symptoms associate with dental apical periodontitis findings in radiographs and represent potential signs of OS. Other causes for OS should also be recognized. To provide adequate treatment, medical professionals should perform a brief assessment of patient’s dental history and status, complete a thorough radiograph evaluation, and utilize microbial findings. Fundamentally, dental professionals play a key role in diagnosing, treating, and, above all, preventing OS.
